# Statistical Picking of Multivariate Waveforms

**DOI:** 10.3390/s22249636

**Published:** 2022-12-08

**Authors:** Nicoletta D’Angelo, Giada Adelfio, Marcello Chiodi, Antonino D’Alessandro

**Affiliations:** 1 Dipartimento di Scienze Economiche, Aziendali e Statistiche, Università degli Studi di Palermo, 90128 Palermo, Italy; 2 Osservatorio Nazionale Terremoti, Istituto Nazionale di Geofisica e Vulcanologia (INGV), 90146 Palermo, Italy

**Keywords:** seismogram, seismic phase picking, change points, changes in variation, cumulative segmentation

## Abstract

In this paper, we propose a new approach based on the fitting of a generalized linear regression model in order to detect points of change in the variance of a multivariate-covariance Gaussian variable, where the variance function is piecewise constant. By applying this new approach to multivariate waveforms, our method provides simultaneous detection of change points in functional time series. The proposed approach can be used as a new picking algorithm in order to automatically identify the arrival times of P- and S-waves in different seismograms that are recording the same seismic event. A seismogram is a record of ground motion at a measuring station as a function of time, and it typically records motions along three orthogonal axes (X, Y, and Z), with the Z-axis being perpendicular to the Earth’s surface and the X- and Y-axes being parallel to the surface and generally oriented in North–South and East–West directions, respectively. The proposed method was tested on a dataset of simulated waveforms in order to capture changes in the performance according to the waveform characteristics. In an application to real seismic data, our results demonstrated the ability of the multivariate algorithm to pick the arrival times in quite noisy waveforms coming from seismic events with low magnitudes.

## 1. Introduction

This paper proposes an extension of a generalized linear-regression-model-based approach to the identification of variance change points [1] of functions that are observed in a fixed domain.

In particular, we describe multivariate signals by proposing a multivariate method that provides the variance change points of multivariate, heteroskedastic Gaussian variables. Although the proposed method can be applied to a wide class of contexts, e.g., the description of multivariate times series, we start from a specific research question that is related to the issue of finding the main phases of seismic curves.

Seismographs provide instrumental observations of earthquakes. A seismogram is a record of ground motion at a measuring station as a function of time. The analysis of seismograms is the basis of experimental seismology, as it is an efficient way to locate the hypocenter of an earthquake and determine its magnitude and source mechanism. A seismogram is constituted by the superposition of different wavefields that propagate up to the Earth’s surface from the hypocenter of the earthquake. The waves that radiate from the seismic source point can be divided into P- and S-waves. P-waves are the fastest of all elastic waves, especially in comparison with S-waves, which are surface and coda waves.

Therefore, seismograms show a characteristic structure: The starting oscillation corresponds to P-waves; in the central part, we generally observe an overlap with S-waves, followed by surface waves in the final part. However, this is an extreme simplification of the structure of a seismogram because, generally, the iteration of wave fields with seismic discontinuities present inside the Earth causes reflection and refraction phenomena, and the origin of new seismic phases can make the seismogram much more complex.

It is important to distinguish between the different seismic phases that appear in seismograms, especially for the location of the hypocenter. For an accurate phase detection and for the interpretation of seismic signals, defining automatic-time seismic phase picking is crucial.

For this purpose, the first issue is the recognition of the different characters of signal and noise in a variety of domains (e.g., amplitude, frequency, and angle features). There is not a single method that ensures consistent picking for any arrival. For instance, the energy approach that is most commonly used cannot be considered in the presence of a high noise level; on the other hand, bandpass filtering does not work when the noise and the signal have almost the same frequency content [2].

The most commonly used automatic picking algorithms were reviewed in [3]. Performance comparisons of different pickers were reported in [4,5,6]. A characteristic function (CF) was introduced in [7,8] through one or several nonlinear transformations of a seismogram, with abrupt increases in the correspondence of the time of occurrence of a seismic wave. The authors of [9] defined a new picking algorithm. In contrast to Allen’s squared envelope function, this CF was sensitive to changes in frequency, amplitude, and phase. Moreover, the distribution density function and some characteristics, such as the main moments (variance, skewness, and kurtosis, which are parameters of higher-order statistics (HOS) [10]) of the seismogram, might characterize their statistical properties. Finally, the so-called autoregressive-Akaike-information-criterion picker (AR-AIC) proposed by [4] was based on the work by [11,12,13,14].

Following [1], in [15], a univariate picking method based on the detection of a signal’s changes in variance was proposed, taking advantage of a formulation of the investigated problem with a generalized linear model, which is relevant for the analysis of changes in the variations of single waveforms.

In [16], a new picking algorithm for the automatic determination of a seismic wave’s time of onset was tested on a simulated waveform dataset in order to capture the variations in performance due to the characteristics of the simulated waveform. Then, in [17], the validity and robustness of the picking algorithm were tested on both synthetic seismograms and real data. In particular, seismic events of different magnitudes (2–5) recorded at different source distances (10–250 km) were simulated, and the results obtained were compared with those obtained by applying the conventional STA/LTA picking algorithm. Although both algorithms gave similar results in the simulations, the proposed algorithm resulted in higher flexibility and greater automation capabilities, as shown in the analysis of the real data.

In fact, the proposed picking algorithm did not require a testing or optimization phase and could be very effective for the routine analysis of earthquakes in new seismic networks or in areas in which the seismic characteristics are unknown.

In these papers, only the vertical component of the recorded seismic event was analyzed, and the main results stated that the algorithm performed best as the distance from the nearest seismic station that recorded the earthquake decreased and the magnitude of the seismic event increased. This is an expected result, as, of course, the shape of the waveform is strictly related to those characteristics of seismic events.

Therefore, motivated by the need for an algorithm that is able to pick arrival times in even more challenging settings, which turned out to be when waveforms were generated by seismic events with a low magnitude and were far from the nearest station that first recorded the event, we intended to use information from other detected components of seismic events to improve the fit of the model and, thus, the detection of seismic phases.

Hence, in this paper, we propose a multivariate automatic approach based on a generalized linear regression model for the detection of change points via a very efficient computational procedure.

We tested the performance of the proposed algorithm by simulating the locations of earthquakes with different magnitudes, which were assumed to be detected at variable distances from the nearest seismic station. This allowed us to highlight the most appropriate scenarios for the multivariate algorithm. To highlight its advantages, we further compared the results of the multivariate picking to those of its univariate counterpart. Finally, we also present an application with real data.

All of the codes were written in the R software language [18] and are available from the authors.

The structure of this paper is as follows. Section 2 proposes the model for the statistical picking of multivariate waveforms, and in Section 3, its performance is assessed through a simulation study. Furthermore, an application to real data is presented in Section 4. Finally, conclusions are drawn in Section 5.

## 2. Moving from Changes in Mean to Changes in Variance

Given ${\left\{\left(x_{i},Y_{i}\right)\right\}}_{i}^{n}$, where $Y_{i}$ is the outcome and $x_{i}$ is the observed explanatory variable for the *i*th sampled unit with i=1,⋯,n, let us assume that $Y_{i}=\mu_{i}+\epsilon_{i}$ with $\mu_{i}$ is, for instance, a sinusoidal function representing the observed signal, and $\epsilon_{i}\overset{id}{∼}N\left(0,\sigma_{i}^{2}\right)$ with $\sigma_{i}^{2}$ is a variance function that is approximated by a piecewise constant regression function with $K_{0}+1$ segments.

Using the generalized linear model formulation, the test for the change in variance of a Gaussian random variable sequence can be equivalently transformed into a test for the change point of the rate parameter of a gamma sequence. Therefore, the changes in the variance of the original sequence are individuated in an attempt to look for changes in the mean of the residuals of the fitted linear model.

This represents a generalized reformulation of the approach of the *cumSeg* model proposed in [19], where a least-squares approach was used to detect multiple change points in genomic sequences. In fact, this paper shows a novel approach to obtaining estimates of the number and location of variations when the dispersion function is described by a piecewise constant function with unknown jump points. In particular, we also account for the multivariate nature of data while looking for change points in the variance of multivariate curves, extending the results of [1].

Let $Y_{i}=\left(Y_{i1},\cdots ,Y_{ij},\cdots ,Y_{id}\right)$ be the vector of the multivariate response for the *i*th unit, i=1,⋯,n, with $Y_{i}\in R^{d},d\geq 1$. Assume that $Y_{ij}$, which is denoted as $Y_{i}$ for simplicity, has an exponential family distribution with the following density:

(1) $$f\left(Y_{i}\right)=\exp\left[\left\{Y_{i}\mu_{i}-b\left(\mu_{i}\right)\right\}/a\left(\phi_{i}\right)+c\left(Y_{i},\phi_{i}\right)\right]\;\;i=1,\ldots ,n$$

where *a*, *b*, and *c* are known functions that characterize the distribution and $\phi_{i}$ is a known dispersion parameter (refer to [20] for the multivariate formalism). Given *g* as the link function, the model for changes in $g(\mu_{i})$ can be formulated as follows:

(2) $$g\left(\mu_{i}\right)=\beta_{1}+\delta_{1}I\left(x_{i}>\psi_{1}\right)+\ldots +\delta_{K_{0}}I\left(x_{i}>\psi_{K_{0}}\right),$$

where *g* is the link function, $x_{i}$ is a broken-line covariate, and the ψs represent $K_{0}$, or the locations of the changes (*change points*). $\beta_{1}$ represents the mean level for $x_{i}<\psi_{1}$, while $\delta_{k}$ is the vector of the differences in the mean levels at the change points. We refer the reader to [1,19] for more details on the fitting procedure. In the multivariate case, we developed a model for modeling the variance change points in multivariate signals; this was still based on the estimation of the model (2), where the response was the squared sum of Gaussian heteroscedastic variables. Following the suggestion of [1,21], once the mean signal ${\hat{Y}}_{i}$ was estimated with a standard smoothing procedure, e.g., a cubic smoothing spline, we modeled the squared studentized residuals $s_{i}={\left(Y_{i}-{\hat{Y}}_{i}\right)}^{2}/w_{i}$ with weights $w_{i}=1-h_{i}$, where $h_{i}$ is the *i*th diagonal element of the hat matrix *H*, as the response of the model (2). In the uni-dimensional case, the chosen distribution model was the Gamma one because of the strict positiveness and typically skewed dispersed nature of residuals.

Extending to a multivariate context, the Dirichlet multinomial distribution models multivariate data that exhibit over-dispersion [22]. In particular, the Dirichlet multinomial is a multivariate extension of the beta–binomial distribution, as the multinomial and Dirichlet distributions are multivariate versions of the binomial distribution and beta distribution, respectively. In this case, the density of the *d*-dimensional vector Y with the parameter $\alpha =\left(\alpha_{1},\cdots ,\alpha_{d}\right),\;\alpha_{j}>0,$ is

(3) $$P\left(Y|\alpha \right)=C_{Y_{1},\cdots ,Y_{d}}^{m}\prod_{j=1}^{d}\frac{\Gamma \left(\alpha_{j}+Y_{j}\right)\Gamma \left(\sum_{j^{\prime }=1}^{d}\alpha_{j}^{\prime }\right)}{\Gamma \left(\alpha_{j}\right)\Gamma \left(\sum_{j^{\prime }=1}^{d}\alpha_{j}^{\prime }+\sum_{j^{\prime }=1}^{d}Y_{j}^{\prime }\right)},$$

where $m=\sum_{j=1}^{d}Y_{j}$. Here, $C_{k}^{n}$, often read as ‘*n* choosing *k*’, refers to the number of *k* combinations from a set of *n* elements.

The structure of the algorithm then follows that proposed in Adelfio [1], with the only difference being that in the present approach, the squared residuals are modeled as responses of the Dirichlet multinomial regression model, and the tools of the MGLM package [23] in the R software [18] were used.

Starting from the model (2), a user-fixed number of $K^{*}$ change points were estimated, say ${\hat{\psi }}^{*}$. Then, the two change points ${\hat{\psi }}_{1}$ and ${\hat{\psi }}_{2}$, corresponding to the arrival of the first P-wave and the arrival of the first S-wave, were selected among the $K^{*}$ points through the procedure denoted as *changepost*, and this is reported in Algorithm 1.
**Algorithm 1***changepost***Require:**${\hat{\psi }}^{*}=\left\{{\hat{\psi }}_{1}^{*},\cdots ,{\hat{\psi }}_{K^{*}}^{*}\right\}$; $s_{i}={\left(Y_{i}-{\hat{Y}}_{i}\right)}^{2}/w_{i}$; $S=\{s_{1},\cdots ,s_{n}\}$; start=1; end=n**Ensure:**$\hat{\psi }$    1:**for**$\left(i\;in\;1:K^{*}\right)$**do**2:    **if** (i==1) **then**3:        $ratio\left[i\right]\leftarrow \frac{\mathrm{var}(S\left[start:{\hat{\psi }}_{i}^{*}\right])}{\mathrm{var}(S\left[start:{\hat{\psi }}_{i+1}^{*}\right])}$4:    **end if**5:    **if** $\left(1<i<K^{*}\right)$ **then**6:        $ratio\left[i\right]\leftarrow \frac{\mathrm{var}(S\left[{\hat{\psi }}_{i-1}^{*}:{\hat{\psi }}_{i}^{*}\right])}{\mathrm{var}(S\left[{\hat{\psi }}_{i-1}^{*}:{\hat{\psi }}_{i+1}^{*}\right])}$7:    **end if**8:    **if** $\left(i==K^{*}\right)$ **then**9:        $ratio\left[i\right]\leftarrow \frac{\mathrm{var}(S\left[{\hat{\psi }}_{i-1}^{*}:{\hat{\psi }}_{i}^{*}\right])}{\mathrm{var}(S\left[{\hat{\psi }}_{i-1}^{*}:end\right])}$10:    **end if**11:**end for**12:$ratio\left[ratio<1\right]\leftarrow {\left(ratio\left[ratio<1\right]\right)}^{-1}$13:$\hat{\psi }\leftarrow {\hat{\psi }}^{*}\left[\mathrm{which}\left(\mathrm{rank}\left(ratio\right)>K^{*}-2\right)\right]$

Basically, we computed the ratios between the variances of the subsequent phases identified by the $K^{*}$ estimated change points. The two relevant change points $\hat{\psi }$ were selected in correspondence with the highest variance ratios. Of course, the larger the fixed number of change points $K^{*}$ estimated by the model (2) is, the larger the set of the two most potentially relevant change points selected by Algorithm 1 will be. Indeed, once the change points were selected by the proposed algorithm, we chose to distinguish those associated with the P- and S-waves based on their arrival times. Nevertheless, the first arrival time in seismograms is commonly defined as the arrival of the first P-wave.

For the problem of estimating the correct number of change points in segmented regression models, we refer the reader to [24].

## 3. Simulation Study: Multivariate Sequence Waveforms

Waveforms were simulated with different characteristics, such as different magnitude values and different distances from the seismic station that first recorded the event. In particular, we resorted to the simulation approach from [25], which involved summing the recorded seismograms from small earthquakes in the past with suitable time shifts and generating high-frequency seismograms. The dataset was made of 1000 waveforms of 300 s each (with a 250 Hz sampling step). Moreover, for each earthquake, the noise was simulated and added to the original sequence. Using a combination of the categories derived from the quantiles of the univariate distributions of the two variables (magnitude and distance from the station), we generated 16 alternative waveform scenarios. Consequently, a different number of waveforms was involved in each scenario, as shown in Table 1.

**Figure 1:**
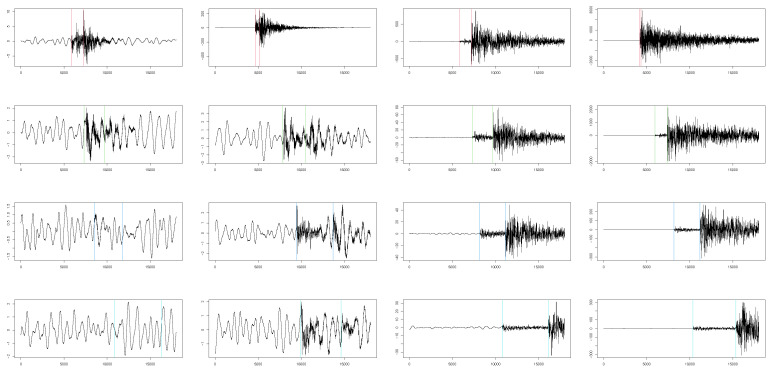
An example of the simulated waveforms for each of the discussed scenarios and the true arrival times in seconds multiplied by the sampling step in hertz. *In the columns*: magnitude levels from Table 1, increasing from left to right. *In the rows*: distance from the nearest seismic station from Table 1, increasing from top to bottom.

The relevant change points were defined as the true times of arrival of the first P- and S-waves, which are denoted as $\psi_{1}$ and $\psi_{2}$, respectively (i.e., $K_{0}=2$ and $K^{*}=10$). Figure 1 displays an example of waveforms for each scenario. The vertical lines show the true arrival times. From this representation, the relationship between the shape of the waveform and the characteristics of the seismic event is evident.

We generated all three waveform components, namely, North–South, East–West, and vertical, for each earthquake. In this paper, we chose to report only the results for the two horizontal components, i.e., North–South and East–West. Furthermore, we analyzed the first 1/3 of the waveforms, as the remaining time period of the waveforms surely did not contain the true change points. Therefore, the rest of the waveforms would not contribute to the detection of the change points, but would only make the computation heavier.

Table 2 shows the empirical means and the mean squared errors in round brackets for the two horizontal components in each scenario. The reported NAs denote when no arrival times were found in the analyzed waveform.

A relevant advantage of the proposed multivariate picking method is the unique estimation of the change points over the two components, since the change points estimated along the two components were very close to each other.

The multivariate algorithm succeeded in picking the arrival times equally in each scenario. This represents a further advantage of the multivariate algorithm with respect to its univariate counterpart. Indeed, the preliminary experiments in [16] showed that the univariate algorithm achieved the best performance with decreasing distances (from the nearest seismic station that recorded the event) and with increasing magnitude. NAs occurred often when the distance is large and the magnitude was small, indicating scenarios in which the P- and S-waves were basically indiscernible from the noise. In those cases, the change points were not easily estimable. To make the results comparable, we ran the simulations with the univariate picking algorithm with the same maximum number of estimated change points $K^{*}$, which was equal to 10, and we computed the mean squared errors of the multivariate estimates as the mean over the estimates for the two components, divided by their standard deviations. As shown in Table 2, the NAs (no estimated arrival times) for the univariate case strongly depended on the characteristics of the waveforms, and they failed completely to identify any change points in some scenarios, which were those with the largest distances from the station and with the lowest magnitudes.

Furthermore, by looking at the mean squared errors, it appears evident that the univariate picking method was the best in detecting the first arrival time in some specific scenarios, while the second arrival time was detected better overall by the multivariate picking method. Indeed, we could not identify relevant differences among the different scenarios. On the contrary, the mean squared errors of the univariate picking method increased as the distance from the station and the magnitude increased.

## 4. Application

This section describes the application of the proposed methodology to a real dataset from the seismic sequence that occurred in L’Aquila (Italy) in 2009. The seismic events of the L’Aquila cluster were selected while considering all of the seismic events that fell in a circular area within a radius of 10 km, centered on the epicenter of the main shock in the city of L’Aquila with a magnitude of 5.9. This event, which took place on April 6 at 03:32, had the following hypocentral coordinates: latitude = 42.349 N, longitude = 13.38 E, hypocentral depth = 8.3 km. In order to collect the events with the highest signal-to-noise ratios that were simultaneously recorded by a large number of stations, a threshold magnitude of 3 was set. Events with a magnitude lower than this threshold were not taken into consideration. The only 3D broadband stations that were selected were those that recorded all of the seismic events identified with the above criteria. The final dataset was made up of 80 earthquakes recorded by 12 seismic stations. The three components of motion to which they referred (up–down, North–South, and East–West) were available for 2880 waveforms in total. Each waveform was sampled at 100 Hz, and the total length of each waveform was 100 s (10,000 samples). To increase the signal-to-noise ratio, a bandpass filter was preliminary applied, with a frequency (FIR 6) between 1 and 35 Hz. That operation was necessary in order to discard frequencies that were related to electronic and anthropic noise, which were clearly not parts of the seismic signals. Further, a normalization with respect to the waveform’s maximum amplitude was applied. This particular dataset represents a complex scenario in which the univariate algorithm failed to pick any arrival times.

We only considered events coming from the BSSO—Busso station (Italy); thus, we selected waveforms with different shapes. Having selected only events falling in a circular area with a radius of 10 km around the main shock, the distances from the seismic station were similar. The BSSO station had the following coordinates: latitude = 41.5461 N and longitude = 14.5938 E. This made it approximately 134.3 km distant from the L’Aquila main shock. Furthermore, all of the seismic events exhibited magnitude levels between 3 and 4.1, making this scenario quite challenging for the selection of the change points.

**Figure 2:**
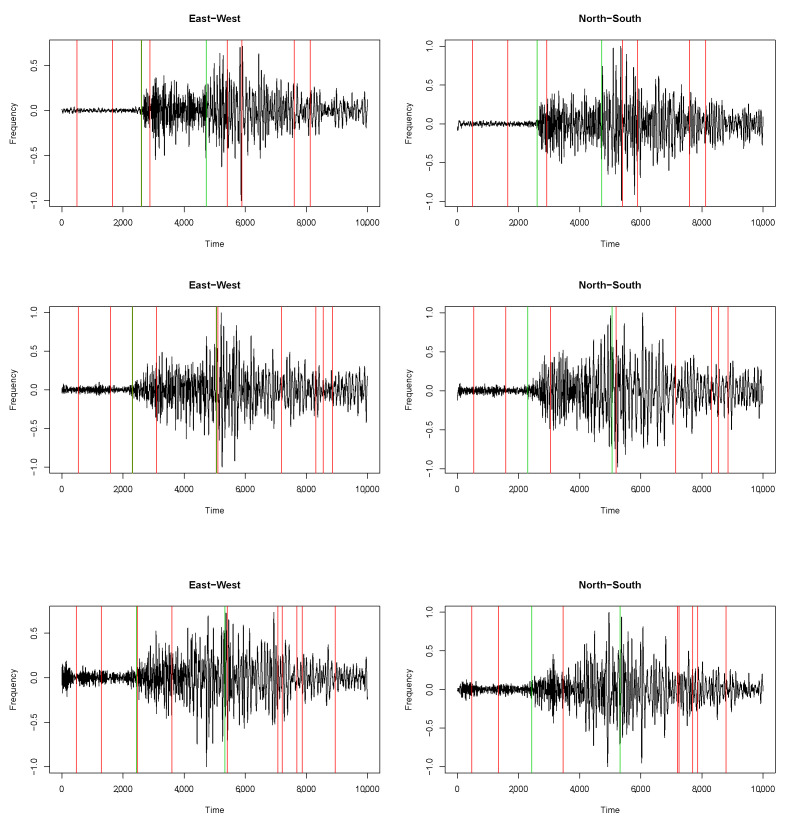
Waveforms of three earthquakes in the L’Aquila seismic sequence that occurred in 2009, detected by the BSSO—Busso seismic station (horizontal components). Red vertical lines: the $K^{*}=10$ change points detected by the multivariate algorithm. Green vertical lines: the two change points selected by Algorithm 1 among the $K^{*}$s.

Figure 2 shows three waveforms belonging to the selected seismic sequence. The red vertical lines indicate the $K^{*}=10$ change points estimated by the multivariate algorithm. The green lines indicate the two change points selected by Algorithm 1, which most likely represent the true P- and S-waves’ arrival times. Overall, the proposed multivariate procedure succeeded in picking the arrival times in 23 out of the 80 seismic events. The univariate counterpart only picked 3 times out of 80, though it started from $K^{*}=10$.

This represents an important result, as we showed that the multivariate algorithm could detect arrival times in quite complex waveforms that came from seismic events with considerably low magnitudes. Indeed, in seismic sequences, such as that which occurred in L’Aquila, the majority of the detected waveforms come from events with lower magnitudes (and, consequently, complex waveforms). The simultaneous information provided by the two components helps in the identification of the arrival times in scenarios in which only one component is not sufficient.

## 5. Conclusions

In this paper, we developed an efficient and simple method for detecting unknown change points in multivariate regression models with a piecewise constant variance function.

This approach can be seen as a generalization of the *cumSeg* procedure from [19] for any regression model and of the approach proposed in [1], since we tested for changes in the variance of multivariate signals.

The multivariate version of the picking algorithm from [17] was tested through simulations. With this aim, we simulated waveforms with different magnitudes and distances from the nearest seismic station.

The results showed an improvement in the picking of the seismic phases; therefore, the exploitation of other components’ information is crucial for the better detection of such phases. With particular reference to the analysis of real data presented in this paper, the proposed multivariate procedure succeeded in picking the arrival times correctly in 23 out of the 80 seismic events, that is, it had a success rate of about 30% of the cases, unlike the univariate method, which picked values for about 4% of the cases. This constitutes a great improvement. Nevertheless, we are aware that this percentage is quite low, and therefore, we plan to test the performance of the algorithm with additional studies of real data.

The most relevant result of the multivariate version of the picking algorithm was its ability to estimate the times of arrivals regardless of the waveforms’ shape. Apparently, the information provided by the two components that were simultaneously analyzed counterbalanced the need for a less complex shape of the waveforms, though this was necessary for the univariate picking method in order to correctly identify the arrival times.

A further advantage of the multivariate procedure in comparison with its univariate counterpart is its ability to provide a unique estimate of the change points over the two components used in the fitting procedure.

In the manual picking of P- and S-waves, it is a well-established practice to pick the P-waves’ arrival on the vertical component, while the S-waves arrive on one of the horizontal components. This is mainly because P-waves are better discriminated on the vertical component, while horizontally polarized S-waves are more easily recorded on the horizontal components. We decided not to use this observation at this stage of our proposal in order to make the algorithm generalizable to other application fields in which testing a change in variance could be crucial. Some fields of application include stock market records, the analysis of blood flows, etc. For this reason, we believe that one of the main advantages of the proposed methodology is its broad application field. This, of course, is related to its ability to detect multiple change points in a variance function; thus it is also applicable to jump-points via a computationally efficient procedure (see, for instance, Muggeo and Adelfio [19], Huang et al. [26], and Tibshirani and Wang [27] for different applications using mean change point detection approaches).

## Figures and Tables

**Table 1:** Number of waveforms selected for the 16 simulated scenarios.

		Km		
**M**	**(0–62]**	**(62–125]**	**(125–187]**	**(187–250]**
(2.00–2.75]	63	63	71	66
(2.75–3.50]	57	63	63	84
(3.50–4.25]	54	47	68	67
(4.25–5.00]	60	56	68	50

**Table 2:** Empirical means and mean squared error values (in round brackets) for the two relevant change points over the two horizontal components (North–South and East–West) in each simulated scenario, expressed in seconds.

	True		Multivariate		Univariate			
					**North-South**		**East-West**	
	$\psi_{1}$	$\psi_{2}$	$\psi_{1}$	$\psi_{2}$	$\psi_{1}$	$\psi_{2}$	$\psi_{1}$	$\psi_{2}$
(62–125]								
(2.00–2.75]	24.66	28.01	23.00(0.37)	28.05(0.09)	25.73(3.46)	29.53(1.65)	25.86(1.16)	30.04(1.13)
(2.75–3.50]	25.21	28.97	24.55(0.83)	31.83(0.10)	25.80(0.11)	31.30(2.01)	25.72(2.54)	30.89(3.34)
(3.50–4.25]	25.68	29.77	25.39(0.45)	29.69(0.06)	26.64(35.78)	30.54(0.30)	25.88(41.67)	30.66(14.41)
(4.25–5.00]	24.61	27.94	23.12(0.54)	25.90(0.07)	25.84(0.75)	26.84(0.73)	25.75(16.45)	26.66(22.32)
(62–125]								
(2.00–2.75]	34.29	44.57	33.99(0.20)	45.11(0.12)	37.54(50.71)	44.56(77.22)	38.69(10.01)	45.03(17.11)
(2.75–3.50]	34.63	45.17	36.00(0.51)	46.66(0.17)	34.79(5.70)	44.98(0.18)	34.44(10.20)	45.04(10.98)
(3.50–4.25]	34.41	44.78	34.23(0.60)	44.95(0.23)	33.98(0.23)	41.60(0.28)	33.95(68.31)	41.98(59.88)
(4.25–5.00]	34.19	44.41	33.09(0.40)	45.07(0.16)	33.65(0.46)	42.62(0.49)	33.89(70.87)	43.51(61.40)
(125–187]								
(2.00–2.75]	43.63	61.11	45.81(0.41)	61.59(0.14)	NA(NA)	NA(NA)	NA(NA)	NA(NA)
(2.75–3.50]	43.62	61.16	43.66(1.73)	61.58(0.27)	42.99(8.78)	59.83(0.24)	43.40(41.70)	57.54(45.79)
(3.50–4.25]	43.96	61.74	43.55(0.59)	63.41(0.44)	43.70(0.17)	64.33(0.17)	43.68(70.00)	65.26(89.16)
(4.25–5.00]	43.63	61.08	43.37(0.87)	63.54(0.47)	43.14(0.28)	62.22(0.31)	43.11(74.97)	62.88(66.31)
(187–250]								
(2.00–2.75]	51.33	75.93	50.90(0.62)	75.53(0.62)	NA(NA)	NA(NA)	NA(NA)	NA(NA)
(2.75–3.50]	51.58	76.38	51.35(0.95)	74.00(0.30)	50.42(0.29)	62.83(24.51)	49.78(153.87)	60.60(314.60)
(3.50–4.25]	51.32	75.91	51.32(0.88)	76.28(0.66)	51.15(7.12)	70.82(0.12)	51.32(122.44)	73.75(77.75)
(4.25–5.00]	51.62	76.45	49.84(0.44)	77.96(0.66)	50.51(0.24)	75.10(0.32)	50.95(24.28)	74.90(39.55)

## Data Availability

Data are available from the first author.
